# Immersive Simulation Training: Remote Patient Monitoring of Complex Older Adults

**DOI:** 10.1093/geroni/igaf122.4135

**Published:** 2025-12-31

**Authors:** David Picella, Sanggon Nam, Lowell Renold

**Affiliations:** Azusa Pacific University, Azusa, California, United States; Azusa Pacific University, Azusa, California, United States; Azusa Pacific University, Azusa, California, United States

## Abstract

In value-based geriatric care, building practical skills in managing chronic conditions is vital. This pilot study assesses an immersive training program using Remote Patient Monitoring (RPM) to enhance nurse practitioner (NP) students’ perceptions and skills. Eight NP students participated in a 10-day immersion simulation, embodying the patient experience by using RPM devices (blood pressure monitors, glucose meters, weight scales, pulse oximeters) to report daily vitals via a secure portal. They also simulated medication adherence to experience challenges of managing complex regimens, through hands-on patient role immersion. Pre- and post-perceptions were measured on a 5-point Likert scale, analyzed via Wilcoxon Signed-Rank and Paired Samples T-Tests. Qualitative responses were thematically analyzed for discoveries, perspective changes, and therapeutic challenges. Quantitative findings showed a significant decrease in perceived RPM cost burden (Wilcoxon p = 0.046; T-Test p = 0.033; Pre-Mean=3.38, Post-Mean=2.88), with no significant shifts in RPM’s importance, liability, time consumption, feasibility, or medication ease (p > 0.05). Qualitatively, themes emerged around heightened understanding of older adults’ daily struggles with technology, adherence, and complex regimens; appreciation for RPM in averting hospitalizations; and calls for interprofessional collaboration. This pilot demonstrates that RPM simulation training enhances NP students’ understanding of geriatric care complexities, critical for value-based care. Interprofessional educators and clinicians can leverage such training to improve chronic disease management, emphasizing simplified regimens and team-based approaches to enhance patient outcomes.

